# Field Sampling of *Zaprionus indianus* in the Eastern United States in 2023-2024

**DOI:** 10.17912/micropub.biology.001707

**Published:** 2025-07-17

**Authors:** Weston J. Gray, Megan Stephenson, Alan O. Bergland, Priscilla A. Erickson

**Affiliations:** 1 Biology, University of Richmond, Richmond, VA, USA; 2 Biology, University of Virginia, Charlottesville, VA, USA

## Abstract

We conducted surveys for the invasive drosophilid *Zaprionus indianus* in the eastern United States in 2023 and 2024. We found no latitudinal trends in *Z. indianus* abundance, and the northern boundary for *Z. indianus* was variable between years. *Z. indianus* was rare in central and northern Florida in the spring, was not present on early season berry crops in Virginia, and had a restricted temperate growing season compared to other drosophilids, providing further support for the species’ yearly recolonization of temperate areas. *Z. indianus* was also observed on native fruits in a natural area in Virginia.

**Figure 1. Z. indianus survey results from 2023 and 2024:**
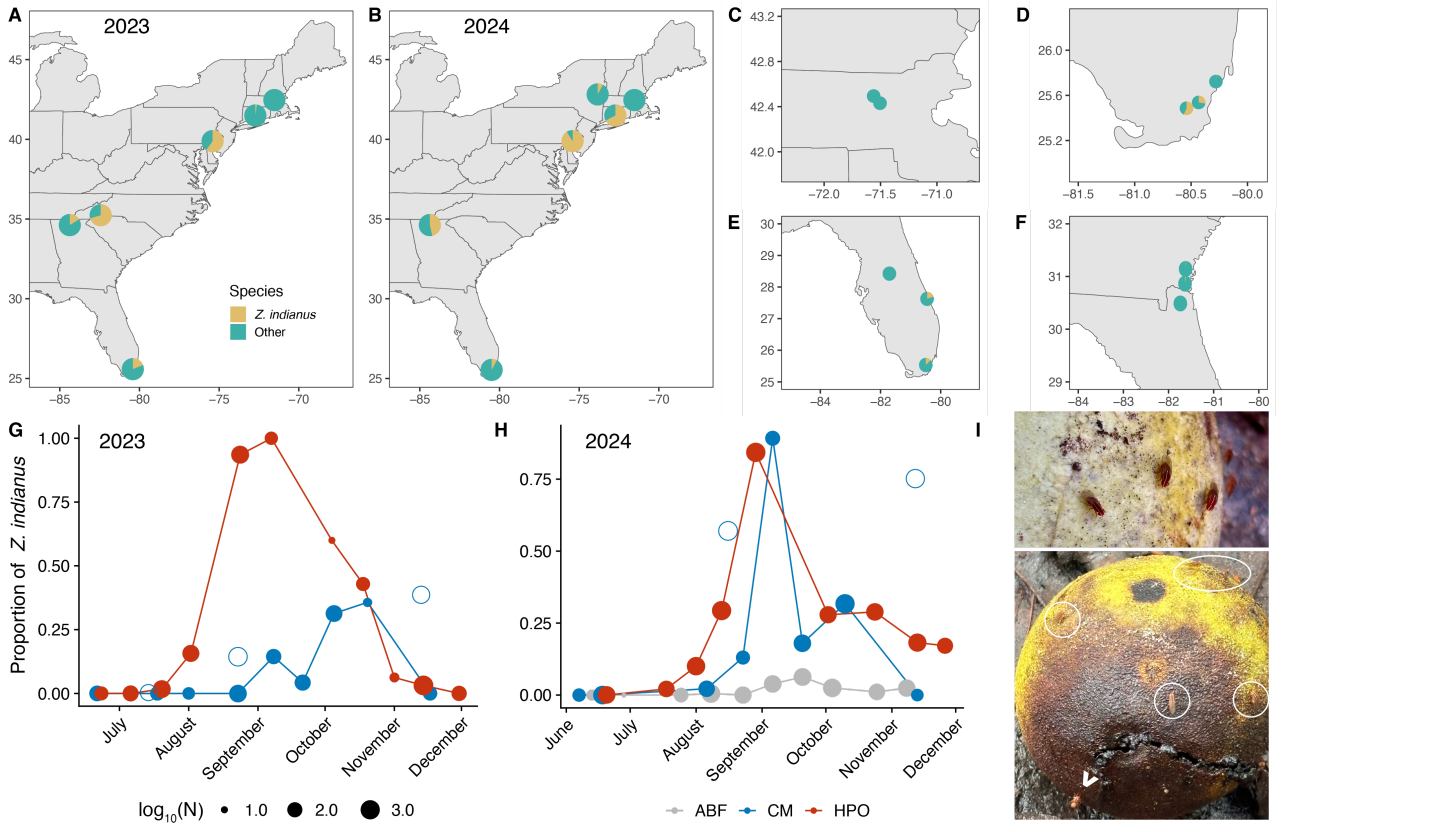
All data are shown as the proportion of *Z. indianus* relative to other drosophilids from collections in 2023 and 2024. A-B) Results of latitudinal sampling from 2023 (A) and 2024 (B). C) No *Z. indianus* were collected at two orchards in Massachusetts in either year. D) *Z. indianus* relative abundance varied between sites in southern Florida in November 2023. E-F) Collections conducted in Florida and southern Georgia in March 2024 (E) and May 2024 (F). G-H) Virginia sampling results during the 2023 (G) and 2024 (H) growing seasons. Point size is scaled to the total number of drosophilids captured. Open circles indicate Carter Mountain (CM) collections using nets and aspirators, while the other CM collections (closed circles) were conducted via trapping. HPO=Hanover Peach Orchard; ABF = Agriberry Farm. I) Incidental observation of *Z. indianus* on fallen fruit of *Asimina triloba* (pawpaw, top) and drupes of *Juglans nigra* (black walnut, bottom) in a natural environment in Richmond, VA. *Z. indianus* are circled; arrowhead points to a copulating pair.

## Description

One of the most biologically damaging consequences of globalization is the introduction and spread of invasive species to foreign environments, which can cause irreparable damage to natural ecosystems and inflict severe economic costs (Mooney & Cleland, 2001; Holmes et al., 2009; Oliveira et al., 2013). *Zaprionus indianus* , the African fig fly, is an invasive drosophilid native to tropical Africa that has since spread to India, the Middle East, both North and South America, and to a limited extent Europe (Vilela, 1999; Yassin et al., 2008; Al-Jboory & Katbeh-Bader, 2012; EFSA Panel on Plant Health (PLH) et al., 2022; Nagy et al., 2025). In the western hemisphere, *Z. indianus* was first reported in Brazil in 1999 and subsequently spread across the continent and into North America (Vilela, 1999; Goñi et al., 2001; Van der Linde et al., 2006; Markow et al., 2014). *Z. indianus* was first identified in Florida in 2005 (Van der Linde et al., 2006), was reported in Virginia in 2012 (Pfeiffer et al., 2019), and has since been detected as far north as Minnesota, Quebec, and Ontario (Renkema et al., 2013; Holle et al., 2018).

*Z. indianus* often dominates local drosophilid communities, especially during warmer months, and negatively impacts their diversity (Tidon et al., 2003; Silva et al., 2005; Roque et al., 2017; Pfeiffer et al., 2019; Rakes et al., 2023). Furthermore, *Z. indianus* is an agricultural pest and poses a potential economic threat for fig and berry production, as it is capable of ovipositing on healthy fruits prior to harvest, leading to larval damage of the ripening fruit (Stein et al., 2003; Oliveira et al., 2013; Bernardi et al., 2017; Allori Stazzonelli et al., 2023; EFSA Panel on Plant Health (PLH) et al., 2022). As such, efforts to track the dynamics of its ongoing invasion play an important role in developing effective management strategies and understanding the intricacies of its invasion biology.

We surveyed *Z. indianus* in the eastern United States along a latitudinal transect spanning Florida to Massachusetts in the fall of 2023 and 2024. *Z. indianus* was generally common and widespread; the species comprised over 50% of the total drosophilids collected at many sites ( Figure 1A, 1B, & 1D). Our findings show high variability between localities, as surveys conducted at nearby locations on the same day produced considerably different proportions of *Z. indianus* ( Figure 1D, Rakes et al. 2023). However, some of this variability is likely a result of conducting adjacent sampling efforts at different times of day, since fly activity may vary due to differences in temperature or circadian rhythms. Unlike our sampling from 2022 (Rakes et al., 2023), we did not detect *Z. indianus* in Massachusetts in either 2023 or 2024 ( Figure 1C ), and *Z. indianus* was rare in Connecticut in 2023. However, in 2024, we detected *Z. indianus* in Albany, New York, and their abundance was substantially higher in Connecticut, indicating a reduced range in 2023 relative to 2022 and 2024. Taken together, these findings suggest the northern boundary of its invasion fluctuates yearly. This observation is supported by other studies that have demonstrated similar patterns of annual variability in abundance at higher latitudes (Holle et al., 2018; Dettler et al., 2021; Nagy et al., 2025). Lastly, there was no correlation between abundance and latitude in either 2023 or 2024 (Pearson correlation, t = -0.697, df = 7, P = 0.508, r = -0.255 and t = 0.135, df = 5, P = 0.898, r = 0.06, respectively).

We also conducted temporal sampling of *Z. indianus* at two peach and apple orchards in Virginia in 2023 and 2024. Similar to prior surveys (Rakes et al., 2023), *Z. indianus* did not appear each year until late in the summer, at which point the population expanded quickly within a month and usually became the dominant drosophilid, before slowly decreasing and disappearing before the onset of winter in late November/early December ( Figure 1G & 1H). Sampling method also influenced collections; we often captured a greater proportion of *Z. indianus* by netting and aspirating than using traps at Carter Mountain ( Figure 1G & 1H, compare open and closed circles).

Our findings of a delayed emergence and early disappearance compared to other drosophilids suggest that *Z. indianus* currently undergoes an annual northward recolonization event from southern refugia before being extirpated in the winter (Pfeiffer et al., 2019; Rakes et al., 2023). While we only conducted detailed temporal sampling at a single latitude, studies conducted at localities north of Virginia did not report *Z. indianus* until later in the season than our first detections, suggesting a stepwise progression northwards throughout the growing season (Joshi et al., 2014; Holle et al., 2018). We speculate that this northward expansion is primarily mediated by commercial produce transport as fruits such as peaches and berries ripen earlier at southern latitudes and are shipped north, though we cannot rule out the possibility of natural causes such as wind or migratory flight. Alternatively, *Z. indianus* could overwinter and emerge at different times at different latitudes, but our data and other studies suggest overwintering does not occur in temperate populations. We found few *Z. indianus* in central and northern Florida in the spring, suggesting even mild winters limit population sizes ( Figure 1E & 1F). Another study showed limited populations of *Z. indianus* in northern Florida during the winter and spring, while populations remained abundant in central/southern Florida (Renkema et al., 2018). Likewise, no *Z. indianus* were collected during winter sampling efforts in Memphis, Tennessee (Kohlmeier & Kohlmeier, 2025). Furthermore, although a handful of studies have demonstrated some cold-tolerant traits in *Z. indianus* (Ramniwas et al., 2012; Lavagnino et al., 2020), many others have demonstrated limitations in freezing conditions (Karan et al., 1999; Araripe et al., 2004; Nava et al., 2007; Comeault et al., 2020; Lavagnino et al., 2020). While our findings are consistent with annual recolonization, additional fine-grained temporal sampling and physiological studies are needed to definitively reject the hypothesis of overwintering.

We also monitored a Virginia berry farm in 2024 to determine whether *Z. indianus* were present on berries in the early summer, prior to peaches ripening at other locales later. No *Z. indianus* were detected at the berry farm prior to other locations in Virginia, and they remained uncommon throughout the fall even while abundant at surrounding orchards ( Figure 1H, gray). Previous studies have reported mixed results regarding *Z. indianus* abundances near brambleberries (Joshi et al., 2014; Holle et al., 2018; Dettler et al., 2021; Allori Stazzonelli et al., 2023), and differential use of pesticides could explain variation in abundances between sites (Andreazza et al., 2016; de Oliveira Rios et al., 2024). While our results collectively suggest *Z. indianus* arrives in Virginia later in the summer, the lack of *Z. indianus* on berries earlier in the season may instead be a product of the local environment at the berry farm we sampled, rather than a true reflection of the species’ absence in Virginia at that time. Our finding that *Z. indianus* emerges in Virginia at approximately the same time each year demonstrates relative consistency in the timing of expansion. We suggest that this timing pattern is largely due to the structured seasonality of produce production and transport. However, *Z. indianus* has previously been collected in Virginia as early as June, suggesting additional variables impact the timing of its annual expansion, or that overwintering could occur in some years (Erickson et al., 2025).

Apart from our planned collections, we incidentally found evidence of *Z. indianus* aggregating and copulating on fallen pawpaw ( *Asimina triloba* ) fruits and drupes of the black walnut ( *Juglans nigra* ) in a natural park in Richmond, Virginia ( Figure 1I ). To our knowledge, this is the first report of *Z. indianus* utilizing either species as a host (EPPO, 2025). Further examination will be required to determine whether these native fruits are suitable larval hosts. Previous studies have found *Z. indianus* utilizing native fruit to varying success, suggesting the species is invading both agricultural and native ecosystems (Leão & Tldon, 2004; Castrenza, 2011; Joshi et al., 2014). The lack of commercial produce production or transport at this location suggests that short-range dispersal of *Z. indianus* can occur independently of fruit shipments. Thus, the use of wild hosts in natural areas could permit further range expansion without human assistance and would alter the approach to formulating effective management strategies. Furthermore, this expansion to natural habitats could pose additional threats to native drosophilids, which often find refuge from invasive species in these areas (Tidon et al., 2003; Hochmüller et al., 2010; Liu et al., 2023).

*Z. indianus* can undergo rapid evolution in its invaded range (Comeault et al., 2020, 2021; Erickson et al., 2025; Gray et al., 2025). In the future, the species could potentially evolve cold tolerance adaptations that would allow successful overwintering in temperate areas. Year-round populations could have adverse effects on both the local fly communities and agricultural industry of these regions without the limiting factor of annual extirpation. As such, ongoing monitoring is vital to develop and implement effective strategies to limit impacts on both natural and agricultural systems and to track changes that could worsen its invasive potential.

## Methods

Two orchards in Virginia, Carter Mountain Orchard (37.992, -78.472) and Hanover Peach Orchard (37.570, -77.265), were utilized for temporal collections in 2023 and 2024. Both orchards produced peaches in the summer and apples in the fall, and sampling efforts centered around the fruit in season. We collected approximately every 2-4 weeks from June through late November/early December to track the seasonal fluctuations of *Z. indianus* . In 2024, we sampled at Agriberry Farm (37.726, -77.314) to assess the fruit fly community in Virginia during the early summer on blackberries and raspberries. Latitudinal sampling from Florida to Massachusetts took place each fall in both 2023 and 2024, with most collections conducted in October (see Table for locations and dates). Additional collections were conducted in the spring of 2024 in Florida (primarily citrus) and southern Georgia (blueberries) to estimate the northern limits of the year-round population of *Z. indianus* and the dynamics of its initial range expansion north each year.

We netted over fallen fruit and conducted unbiased aspiration directly off the fruit on the ground. Sampling method choice was dependent on a combination of environmental factors such as temperature, precipitation, and abundance of fallen fruit. Traps were used for all Carter Mountain collections (unless otherwise noted) and as needed at some locations. Traps were constructed from 2 L plastic bottles baited with sliced bananas sprinkled with baker’s yeast and were hung from trees or trellises overnight. Flies were collected from the traps the next day via aspiration. See Rakes et al. 2023 for additional sampling methodology details. Collected flies were anaesthetized with CO _2_ and sorted under a stereomicroscope to determine counts of *Z. indianus* and other drosophilids. *Z. indianus* were photographed with an iPhone on pawpaws and black walnuts at Pony Pasture Park (37.551, -77.517) in Richmond, Virginia on September 19, 2024. A subsequent flood prevented us from returning to collect flies.

Data were processed using *data.table* (Barrett et al., 2024) in R (R Core Team, 2024, v.4.3.3) and figures were created using *ggplot2* (Wickham, 2016) and *cowplot* (Wilke, 2024). We conducted Pearson’s correlation analyses to test for a relationship between latitude and the proportion of *Z. indianus* collected.

## Reagents

**Table:** 

State	Orchard	Date of Collection	Latitude	Longitude	Relative Abundance	Total Collected
NY	Bowman Orchards	10/13/2024	42.817	-73.839	0.08	110
MA	Westward Orchards	10/14/2024	42.494	-71.561	0	198
MA	Westward Orchards	10/10/2023	42.494	-71.561	0	241
MA	Carver Hill Orchard	10/14/2024	42.430	-71.504	0	203
MA	Carver Hill Orchard	10/10/2023	42.430	-71.504	0	200
CT	Lyman Orchards	10/15/2024	41.494	-72.730	0.67	273
CT	Lyman Orchards	10/11/2023	41.494	-72.730	0.02	450
PA	Linvilla Orchards	10/16/2024	39.885	-75.410	0.91	309
PA	Linvilla Orchards	10/12/2023	39.885	-75.410	0.6	636
NC	Sky Top Orchard	9/24/2023	35.240	-82.436	0.7	44
GA	Hillcrest Orchards	10/11/2024	34.620	-84.373	0.47	245
GA	Hillcrest Orchards	9/25/2023	34.620	-84.373	0.16	1271
GA	Oglesby Family Farm	5/7/2024	31.144	-81.621	0.02	65
GA	Morning Belle Farms	5/8/2024	30.864	-81.635	0.03	142
FL	Kent's Blueberry Farm	5/8/2024	30.488	-81.738	0	29
FL	Showcase of Citrus	3/29/2024	28.424	-81.704	0	88
FL	Schacht Groves	3/29/2024	27.626	-80.452	0.2	673
FL	University of Miami	11/11/2023	25.722	-80.280	0.01	529
FL	The Fairchild Farm	11/11/2023	25.537	-80.433	0.28	774
FL	Fruit & Spice Park	10/22/2024	25.535	-80.493	0.08	1480
FL	Fruit & Spice Park	3/27/2024	25.535	-80.493	0.13	512
FL	Schnebly Redland's Winery & Brewery	11/11/2023	25.486	-80.541	0.57	67
